# Optimizing CI Systems for Better Recognition of Soft Speech —the Concept of Broad‐Range Mapping

**DOI:** 10.1002/lio2.70273

**Published:** 2025-10-07

**Authors:** Matthias Hey, Thomas Hocke

**Affiliations:** ^1^ Department of Otorhinolaryngology, Head and Neck Surgery, Audiology University Hospital Schleswig‐Holstein (UKSH), Campus Kiel Kiel Germany; ^2^ Cochlear Hannover Germany

**Keywords:** cochlear implant, loudness growth, quiet speech, ScanFF, soft speech, speech comprehension

## Abstract

**Objectives:**

The individual mapping of cochlear implants (CIs) aims to optimize the user's speech understanding. Recent investigations have shown the importance of soft speech: (1) According to Datalog studies, a large proportion of speech components lies in the range below 60 dB, and (2) soft speech represents a separate category in CI outcome, in addition to supra‐threshold speech and speech in noise. Soft‐speech understanding can be influenced by optimizing T‐values or by global parameters (loudness growth and TSPL in the Nucleus system). This study focussed on improving soft speech below 60 dB by optimizing loudness growth.

**Methods:**

Speech understanding with varying loudness growth in the speech processor CP11 (Cochlear Ltd.) was compared in 20 experienced adult CI users. The mean soft‐speech score based on monosyllabic words at 40 and 50 dB was introduced for quantification.

**Results:**

Six of the 20 patients studied showed significant individual improvement for soft speech when loudness growth was optimized, while none showed a significant decrease under quiet or noisy test conditions.

**Conclusion:**

Actual CI systems offer a broad loudness range of speech understanding. In addition to suprathreshold speech understanding, additional attention should be paid to soft speech, and the result should therefore be confirmed by speech audiometry at low levels.

**Levels of Evidence:**

2.

## 1 Introduction

Mapping of cochlear implants (CIs) is aimed mainly at optimizing suprathreshold speech perception for the users. Some studies [1, 2, 3, 4] have highlighted the importance of near‐threshold mapping and its related speech perception as an additional aspect of CI aftercare. Early in the development of CIs, the loudness growth function (LGF)—the rule according to which the different input signal levels are mapped to degrees of electrical stimulation—was considered to play a role in optimizing speech understanding [5, 6]. However, to our knowledge this parameter has not been in the focus of recent studies. For single electrodes the CI mapping has to be adjusted by basic parameters such as threshold (T) and comfort (C) levels for single electrodes [1, 7, 8]. Once this fundamental requirement is fulfilled, the mapping can be influenced globally by parameters such as threshold sound pressure level (TSPL) [9, 10] (which affects mainly the lower end and to a lesser degree the middle part of the LGF), the comfort sound pressure level (CSPL) [11] (which affects the middle part and the upper end of the LGF), and the general LGF (parametrised in the Nucleus CI system as the “Q‐value”, which affects the steepness of the LGF). Usually, the adjustment of electrode‐specific parameters is part of clinical routine, while the global parameters are relatively seldom adjusted [9, 11].

At our centre, patients' C levels are initially mapped on the basis of ECAP recordings [12, 13, 14, 15]. Following first fit, the T and C levels are further adjusted, on the basis of single‐channel thresholds and automated self‐administered categorical loudness scaling [1, 15, 16, 17]. These measurements are based solely on patients' feedback. Several customized speech‐audiometric procedures represent mandatory supplements within CI mapping. This audiometry‐based mapping (also termed audiometry‐based fitting, ABF) is established in our clinic as well as in others [3, 4, 15, 18]; this is performed not only to describe the progress of the CI patients but also to derive individual guidance from these results for further fine‐tuning and optimisation of the patient's speech‐processor mapping. Therefore, multisyllable and monosyllable tests in quiet, as well sentences‐in‐noise tests [3, 4, 13, 15, 19], are currently established methods and can be applied as soon as the patients are able to complete these various tests. On the basis of this approach, CI users are mapped and trained, and this results in most cases, to the best of our knowledge, in open‐set comprehension [3, 20, 21, 22] of speech at conversational level.

As boundary conditions, we can summarize:

- According to datalog studies, a large proportion of speech components is found in the range below 60 dB [23].
- Near‐threshold speech is a separate category to characterize CI outcome [24], in addition to suprathreshold speech and speech in noise. Therefore, understanding of soft speech needs to be optimized separately.

The latter point was also suggested by results of Hazrati et al. [25]—a good performance in suprathreshold speech recognition does not guarantee equivalent performance with whispered speech. Šrámková et al. [26] found that mean values for soft speech lay between 47 and 42 dB_A_. Yet it should be noted that these results were determined for a distance between mouth and microphone of 30 cm; in contrast, within the social space of 1.2–3.6 m [27] this level of speech can easily drop below 40 dB_SPL_. In some recent studies such low and mid‐range speech levels below 65 dB were additionally investigated [4, 10, 28]. Our study was designed to focus on rather less frequently used approaches to improve low‐ and mid‐level WRS. Near‐threshold speech can be adjusted further by optimizing the T values of each individual electrode and by the global parameters LGF and T‐SPL.

In this study, the influence of the globally acting parameters on speech understanding was investigated. The influence of modified mapping parameters was measured, with the aim of improving soft‐level speech understanding. On the basis of pilot measurements, optimized CI parameter settings of steeper loudness growth with *Q* = 17 for speech processor CP11 were compared with the default parameter *Q* = 20 in a population of experienced CI users. A numerically smaller *Q‐*value corresponds to a steeper LGF, that is, stronger amplification at low levels. Speech audiometry for soft and suprathreshold speech in quiet, as well as sentences in noise, were utilized to characterize the entire range of a CI patient's speech comprehension.

## 2 Methods

### 2.1 Subjects

A group of adult CI users (8 female and 12 male) with postlingual onset of deafness participated in this study. The study was approved by the local Ethics Committee (D 581/22). Written informed consent was obtained from CI users prior to participation. All investigations were conducted in accordance with the ethical standards of the Institutional and National Research Commissions and the 1964 Declaration of Helsinki and its subsequent amendments, or with comparable ethical standards.

Additional inclusion criteria for these participants were the use of a CI24RE, CIx12 or CIx32 cochlear implant (Cochlear Limited, Australia) and of a CP1100 speech processor. Participants had to have a full insertion of the electrode array into the scale tympani. They had to achieve a speech understanding of at least 80% in an initial examination using the Oldenburg Sentence Test (OLSA) in quiet (65 dB_SPL_) in order to complete the assessment of speech recognition threshold (SRT) in noise [29]. Bilateral implantation was not an exclusion criterion, but only one ear per patient was examined in this study (10 right and 10 left).

The mean age of the participants was 50 years (minimum 21 years, maximum 83 years). Participants had a mean CI experience of 11.5 years (minimum 2.4 years, maximum 19.0 years).

### 2.2 Test Procedure

All tests were performed in an acoustically shielded audiometric booth (ISO 8253:227).

For testing speech in quiet, the Freiburg tests [30]—comprising two‐digit numbers and monosyllabic words—were used. These tests were carried out with a computer‐based audiometer (Equinox; Interacoustics, Middelfart, Denmark) with evidENT 3 software (Merz Medizintechnik, Reutlingen, Germany). The Freiburg monosyllabic words were presented frontally at different sound pressure levels. Words from each list were presented in randomized order so as to minimize the repetitive learning effect. In this way, the SRT in quiet was determined applying linear interpolation using Freiburg numbers by measuring the discrimination function in 5 dB steps.

For SRTs in noise, the OLSA was used throughout. The loudspeakers were located 1 m from the patient. The following loudspeaker configurations were used: S0N0 (speech and stationary noise from the front [31, 32]) and S0N90 (speech frontal and fluctuating ICRA5 noise 90° [24, 33] ipsilateral to the CI being examined). The Oldenburg sentences [8] were presented at a constant noise level of 65 dB_SPL_. The noises used were the stationary speech‐simulating Oldenburg noise on the one hand and the fluctuating ICRA noise on the other [12]. For the latter, track no. 5 of the ICRA CD was used, which has the spectral and temporal characteristics of a single male speaker. The SRT was measured by using an adaptive method [5] and was defined as the signal‐to‐noise ratio (SNR) that resulted in 50% correct word understanding. All CI users were accustomed to the adaptive testing procedure, having been tested beforehand three times or more in our routine clinical practice. To minimize any differences arising from the procedural learning effect, additional training was given at the beginning of each test session (30 sentences in quiet at 65 dB_SPL_). The measurement of speech understanding was always monaural.

The measurement protocol defined two phases:

- Pilot measurements (*N* = 5),
- Study measurements (*N* = 20).

### 2.3 Pilot Measurements

In the pilot phase, measurements were performed with five subjects; this included repetition (on the same day) to confirm results. Testing was performed in a single session to determine best speech understanding under the seven sets of test conditions (default map with *Q* = 20, T‐SPL = 25 dB, original T‐profile and additional 6 variations). These results were to serve as the basis for testing of the best CI setting in an expanded group of study participants (*N*_total_ = 20) later on.

In the pilot phase, the influence of mapping parameters on speech understanding at low stimulation levels was investigated. The following settings in the CP1100 processor were used as baseline: ADRO, SNR‐NR and ASC activated, *Q* set at 20, T‐SPL set at 25 dB. The following parameters were varied while all other parameters were kept constant: *Q‐*value and T‐SPL (a) *Q* = 20 and T‐SPL = 15 dB; (b) *Q* = 17 and T‐SPL = 25 dB; (c) *Q* = 17 and T‐SPL = 15 dB; and global adjustment of T‐levels (d) original T‐profile minus 5 current level (CL) or (e) original T‐profile minus 10 CL for *Q* = 20 and T‐SPL = 15 dB. For quantification of speech understanding the SRT of Freiburg numbers and the ‘words correct’ score of Freiburg monosyllabic words in quiet at 40, 50 and 65 dB_SPL_ were used.

During the pilot phase, six combinations of the above parameters were evaluated in a single investigation and were compared with default map settings. The combination of original T‐levels with T‐SPL of 25 dB and a *Q‐*value of 17 showed best speech understanding in 4 of the 5 patients. In the remaining patient this combination gave the second‐best result. Therefore, it was decided to compare default CP11 parameters of T‐SPL 25 dB and *Q‐*value of 20 against the optimized parameter set of T‐SPL 25 dB and *Q‐*value 17.

### 2.4 Study Measurements

In the study phase, measurements were performed with 20 subjects (5 from the pilot measurements participated in both studies, along with 15 additional test subject). However, in this phase (i) only the best fitting parameters selected in the pilot phase were used (T‐SPL 25 dB and *Q‐*value 17) in comparison to default map setting (T‐SPL 25 dB and *Q‐*value 20), and (ii) the tests were performed in two sessions spaced two to 3 weeks apart, to allow subjects to accustom themselves to different signal‐processing algorithms under everyday conditions. In the first session of study measurements, patients were tested using their usual standard *Q‐*value of 20. After an acclimatization phase lasting several weeks, the modified *Q‐*value of 17 was examined.

All patients used the ACE coding strategy with individually adjusted stimulation rate and number of maxima. The individual map parameters (T‐ and C‐levels) of the CI speech processors were used unchanged throughout the study period. Scene classifier with the algorithm ScanFF was used. The other algorithms of the acoustic signal pre‐processing were used at default: ADRO, ASC and SNR‐NR were always activated.

The Freiburg monosyllabic words were measured at sound pressure levels of 40, 50 and 65 dB_SPL_, and determination of speech understanding in stationary and fluctuating noise was carried out.

At each examination appointment in the clinic, the CI speech processors were technically checked. If necessary, system components were replaced.

## 3 Analysis

To determine the relative performances of different sound‐processor settings, paired comparison analyses were performed by using the *t* test, whereby each participant served as his/her own control. In all cases, a significance level of 0.05 was used to determine significance for two‐sided analyses.

Averaged word comprehension is used in the measurement of hearing loss as routinely performed in Germany [34] for evaluation of everyday communication skills and thus for certification of any need for financial support from a patient's health insurance. On the basis of this approach, an averaged word‐comprehension score at levels below 65 dB_SPL_ was introduced, in line with the optimized soft‐speech level used in research: mean of ‘soft speech correct score’ at 40 and 50 dB_SPL_.

Results are presented as box plots. These show median (solid mid‐line), 25th and 75th percentiles (box limits), and 5th and 95th percentiles (whiskers). Mean values are also shown as filled squares. Differences between measures were plotted against the mean of the two measures (Bland & Altman, 2010). Positive differences imply a better result in the optimized setting than in the default speech‐processor setting.

## 4 Results

**FIGURE 1:**
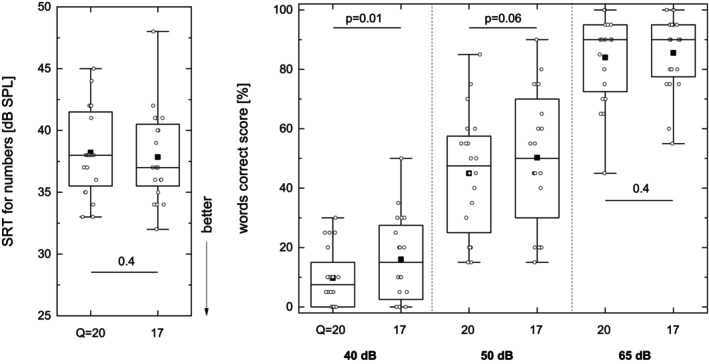
Box plot of SRT for numbers (left) and ‘words correct’ scores for monosyllabic words at different intensities (right). In each case, comparison is made between the default loudness growth function (*Q* = 20) and a steeper function (*Q* = 17).

All 20 patients successfully completed all tests in quiet as well as in noise. Figure 1 shows the results for SRT of Freiburg numbers in quiet as well as monosyllabic words in quiet at various presentation levels from 40 to 65 dB_SPL_. The SRT for Freiburg numbers shows a good correlation with the 500 Hz hearing threshold, with an offset of 18 dB [35]. The Freiburg numbers SRT allows a quick check (less than 5 min) of near‐threshold mapping of CIs, and it showed behavior comparable to that of SRT for sentences in quiet. The main advantage of near‐threshold measurement is achieved by utilizing well‐known two‐digit numbers, which however have the disadvantage of indicating audibility as such rather than actual speech recognition. A tendency for means to show improved speech correct scores for *Q* = 17 was seen, but significant differences was found only for words at 40 dB_SPL_.

**FIGURE 2:**
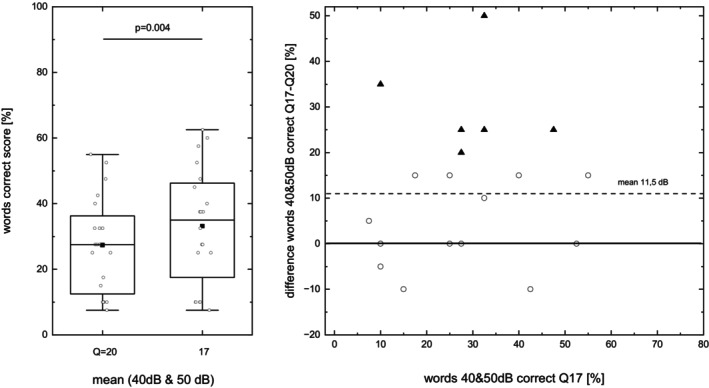
Box plot of mean soft speech ‘words correct’ scores for monosyllabic words at 40 and 50 dB_SPL_ (left) and related individual improvement (right). Comparison between default loudness growth‐function values of *Q* = 20 and (steeper) *Q* = 17. Triangles indicate significant individual improvement.

Averaged soft speech comprehension is shown for the two speech‐processor settings in Figure 2 (left panel), where a significant improvement was seen for *Q* = 17. In addition, Figure 2 (right panel) shows the individual change when switching between the two settings. Positive values show an improvement with the *Q* = 17 setting. Six of the 20 patients investigated showed a statistically significant individual improvement, while none showed a significant decrease in mean ‘words correct’ score [36]. Two of the six patients showing a significant improvement were from the pilot measurement population.

**FIGURE 3:**
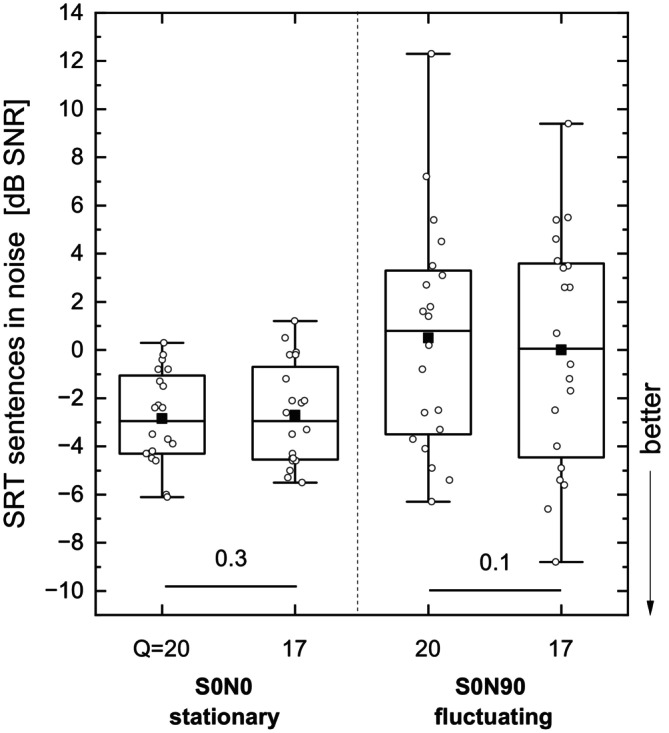
Box plot of SRT speech reception thresholds in noise. Comparison between default loudness growth function values of *Q* = 20 and (steeper) *Q* = 17.

Figure 3 presents the results of the SRT measurements in noise. Mean baseline speech comprehension under S0N0 conditions in stationary noise was −2.8 dB SNR. No significant change was found in stationary or in fluctuating noise at the group level. Combining the results of Figures 2 and 3 leads to the conclusion that none of the six subjects whose quiet score improved individually showed a consistent decrease of SRT in noise.

## 5 Discussion

At the individual level, fine‐tuning of a mapping parameter may potentially improve scores in quiet for soft speech. The results presented here demonstrate this to be the case for the example of LGF. In accordance with some researchers' findings [1, 8, 9]—though in opposition to others [7]—this improvement in quiet was achieved without compromising SRTs in noise. For about a third of our CI users, soft speech understanding improved.

### 5.1 Speech Comprehension for Soft Speech

Our clinical approach of ABF uses loudness‐scaling and speech audiometry of numbers and words to ensure individual optimisation of T levels. There are other methods, such as psychoacoustic approaches, known for fine‐tuning patients' T levels [1, 8]. Since all these approaches yielded comparable performances by CI users for soft speech [1, 23], it can be concluded, firstly, that these methods tend to adjust T levels to an unexpectedly reliable level and, secondly, that near‐threshold mapping facilitates the attainment of improved scores for soft speech without compromising speech‐in‐noise performance.

In an investigation of the previous speech‐processor generation CP10 (Cochlear Ltd.), it was shown that an enlarged acoustic dynamic range is able to improve speech in quiet for levels below 65 dB [10]. This was achieved by lowering the TSPL from 25 to 15 dB_SPL_. In our pilot study, the influence of the widened acoustic dynamic range was compared with changes of loudness growth as well as a global decrease of all T levels. For the CP11 processor used here, the loudness growth showed a positive effect, while a lower T‐SPL did not. Consequently, the present study focused on improving scores for soft speech by optimisation of the global factor loudness growth. Furthermore, these examples of fine‐tuned mapping (T‐level, T‐SPL, *Q‐*value etc.) illustrate the observation that individually optimized CI settings may differ across generations of speech processors. We conclude that each speech processor generation may require detailed optimisation in order to improve even further the CI users' already good speech comprehension.

With regard to clinical effort, it has to be stated that our time‐consuming study protocol limits the clinical application of soft‐speech optimisation by ABF. However, recent studies have shown that there are resources for automatisation in clinical routine: optimized T‐level mapping can be performed by the patient him−/herself within less than an hour, a procedure that probably only has to be performed once [8]. The extensive testing of speech can be automated too. The idea of empowering patients by self‐administered audiometric testing using a tablet was proposed by for example, Philips et al. [37]. Furthermore, the German two‐digit number testing can easily be implemented on tablet versions operated by the CI recipient. Monosyllable scores can be automated once a closed‐set version becomes available. An automated mapping approach [28, 38] potentially contributes to a more extensive version of ABF empowering the CI users themselves.

### 5.2 Broad‐Range Mapping

This study focused on the optimisation of soft speech in quiet. This was motivated by results from an earlier study [24] which identified near‐threshold speech intelligibility as the second factor in characterizing CI outcome. However, soft‐speech audiometry is not yet fully integrated into outcome measurements in CI recipients.

In retrospect, it can be said that in the early years of CI provision the focus was initially on the perception of suprathreshold speech in quiet [39, 40, 41]. Much progress has been made over the last four decades concerning recognition of speech in quiet [14, 42, 43]. With further progress in signal processing in CIs, suprathreshold speech intelligibility in noise has become the next area of interest [43, 44].

Conversation consists of much more than these two suprathreshold aspects. A recent study applying datalogging in cochlear implants has shown that the speech‐related environment of adult CI patients has a maximum in the range of 50–59 dB [23]. This highlights the observation that speech intelligibility at levels below 60 dB_SPL_ is of great relevance for daily life. Furthermore, statistics regarding the lifestyle of adults—for example, in the USA—showed a strong trend toward persons of higher age spending more time alone or with one partner—a situation frequently associated with low noise level and soft‐level speech [45]. This finding about the importance of soft‐level speech is further supported by studies on preferred acoustic environments of hearing‐aid users [46] which contain a considerable portion of speech in the range of 40–60 dB_A_.

The performance of actual CI systems allows the perception of soft speech due to improved microphone technologies [47] and signal‐processing algorithms [48, 49]. Studies with a focus on soft‐speech perception have been described [4, 7, 10, 28].

An additional methodological aspect introduced here is to pay more attention to soft‐speech levels by using the averaged soft‐word recognition score. This is derived from the approach of summed word comprehension across different presentation levels. That value is used in the quantitative assessment of hearing disability in the context of insurance and disability pension issues in Germany [34, 50]. Most remarkably, these attestation rules apply different weights for the different presentation levels.

In about one‐third of the patients investigated here, an improvement in understanding of soft speech was found. The question arises: what about the other patients? Are they already optimally mapped? Or is there another option within the parameter space of a CI system? This should be a subject for further investigation.

Most present‐day CI systems are able to provide reasonable soft‐speech comprehension. This can be achieved by optimizing certain CI‐specific parameters. This *Broad‐Range Mapping* should be added to the established mapping process. It may further decrease the gap between CI users and normal‐hearing persons.

## 6 Current and Future Perspectives

Several studies have reported excellent speech comprehension in quiet for suprathreshold monosyllabic words [22, 51]; audiometric tests show that patients tend increasingly to reach the upper saturation range, so that any further measurable improvement in quality is obviously difficult to attain. Continuing improvement of speech comprehension in noise can be achieved primarily by applying a range of signal‐processing algorithms such as SNR‐NR [47, 52], ForwardFocus [53], ClearVoice [54] or Ambient Noise Reduction [55]. A dedicated algorithm for soft‐level speech, ADRO, was introduced more than two decades ago [48, 56]. It was followed by Whisper [57] and Softvoice [58]. These algorithms must be considered in the context of the specific hardware, including the microphone and its noise floor. Thanks to improved microphone technology and greater computing power, soft speech may be improved still further by signal‐processing techniques. This should influence future CI design.

As this work shows, soft speech can be optimized by accurate T‐level mapping for each electrode [1, 8] and especially by global signal‐processing parameters [9, 10]. It is advisable for CI systems to offer maximum flexibility for a range of parameters improving soft speech. This can be addressed by the CI manufacturer.

During the pilot measurements, the *Q‐*value was found to have the greatest influence on speech comprehension out of the three tested parameters, so it was selected as the main parameter for study measurements. It is possible that a combination of this parameter and the other not‐selected parameters, as well as combinations with other untested parameters, could show even greater improvement. However, testing all combinations of parameters could be too time‐consuming. Therefore, adaptive optimization strategies, such as a Simplex Procedure [59] or a Genetic Algorithm [60], could be used to investigate this aspect more efficiently in future studies.

Another issue that audiologists must address is that, as described above, guiding patients to their best possible CI outcome still requires significant clinical mapping effort. In the future, we may expect a workflow that provides relief of clinical resources by introducing patient empowerment, self‐administered audiometry, and additionally new mapping approaches based on machine learning with a strong link to recent audiological findings. In this way, it may be expected that more patients will be able to benefit from improvements of this kind.

## 7 Conclusion

Actual CI systems make speech understanding possible in a broad range of presentation levels, ranging from suprathreshold speech at 65–80 dB down to levels of 40–50 dB typically found for soft and distant voices. There is a need for supplementary focusing on soft speech, in addition to suprathreshold speech. This is motivated by the facts that (1) soft‐level speech is an inherent and a major part of speech in everyday life, while being of social importance, and (2) soft speech is a separate category in CI outcome.

The advantage of steeper volume growth functions may be specific to the devices used, whereas previous processor generations have other microphone characteristics. For this reason, one recommendation of this study could be to use Q values of 17 by default for the CP11 processors examined. This can also be tested on previous speech processor generations and verified by means of speech tests.

Audiometry‐based fitting has to be underpinned by speech audiometry over a broad range of presentation levels. Broad‐Range Mapping, with its additional focus on soft speech, includes precision T‐level mapping across each electrode and optimizing parameters such as loudness growth or other globally acting factors. The individual optimal parameter set might be different for each patient and should possibly be reconsidered for each processor generation in the presence of ongoing signal processing development. Future research to develop this principle may be expected to reduce the burden on clinicians while at the same time achieving the greatest possible benefit for each patient.

In general, more attention should be paid to soft‐level quiet speech in the future. Soft speech can be optimized without any trade‐off with speech understanding in suprathreshold quiet or in noise. This can be achieved by exploiting to the fullest extent the flexibility of a CI system's mapping parameters and through its individual audiological optimization.

## Conflicts of Interest

Thomas Hocke is an employee of Cochlear Deutschland GmbH. The authors report no other potential or actual conflicts of interest. The authors alone are responsible for the content and writing of this paper.

## Data Availability

The data that support the findings of this study are available from the corresponding author upon reasonable request.
